# Supplementary material for: Accuracy of a Bayesian technique to estimate position and activity of orphan gamma-ray sources by mobile gamma spectrometry: Influence of imprecisions in positioning systems and computational approximations
Source: PLoS One. 2022 Jun 22;17(6):e0268556. doi: 10.1371/journal.pone.0268556 (PMC9216606; doi:10.1371/journal.pone.0268556)
Supplement: Attachment — Submitted filename: Reviewer_comments_20210622.pdf [file pone.0268556.s001.pdf]

Review of the manuscript entitled "*Accuracy of a Bayesian technique to estimate position and activity of orphan gamma-ray sources by mobile gamma spectrometry: Influence of imprecisions in positioning systems and computational approximations*" by Bukartas *et al.*, submitted for publication to PLOS ONE.

**General comment:**

The present paper is the continuation of the author's previous work that was published in PLOS ONE. In this paper, authors investigated the accuracy of their proposed technique for estimating the activity and position of the source that has been lost. The present work is interesting and also important as it involves actual experiment to test the proposed theory. However, there are few issues that needs to be addressed. Please see my comments as attached and make changes to the current version of the manuscript. Considering my comments, I recommend <u>minor revision</u> of the manuscript before being considered for publication in PLOS ONE.

**The comments:**

1) In abstract, "This reduces the computational demands increasing the potential of real time applications of the method". Please re-write.

2) In line 9, "but gives no direct information on the distance to the source or its activity.", the detector is measuring some activity, it is better to refer to "actual activity" of the source, when addressing this.

3) In line 15, "with which", consider changing to "at which".

4) In line 47, "…orphan source the parameters of interest" add a comma after "source", please take care of the punctuation throughout the paper. There are many issues similar to what I pointed out here.

5) In lines 58-59, "vague prior is usually used", do you mean something like an initial guess? Please revise accordingly.

6) In line 59, "large variance", how do you defined this variance, statistically speaking "variance" would only be meaningful when we have a mean (refer to the formula). Please clarify briefly.

7) In lines 60-61, "However, if there is reason to believe that a radioactive source is in the area, it can be assumed that it could be anywhere in the area." Please re-phrase and re-write.

8) In line 64, "Similarly, a Gamma distribution with large shape and rate parameters…", this confusing to me, have you employed Gamma distribution in your model, or you just use this as an example since the distribution takes two parameters? Please clarify.

9) In line 106, "a set of arbitrary initial values of", by "arbitrary" do you mean "guess values"? please revised accordingly.

10) In line 110, “uniform random number between 0 and 1”, how did you generate these random numbers? Was it produced sequentially in your model or it’s an array? How did the seed changes in each iteration? Please explain briefly.

11) In line 126, please define “GNSS”.

12) You have provided a schematic of the vehicle’s path and source locations. It would be nice to get some pictures of your experimental setup, so that the readers can understand how detectors were setup in the vehicle.

13) In Fig. 1, please consider changing the symbol for source location from “×” to something like a dot, as there will be lesser overlap between the symbols.

14) In line 149, “…at a steady speed of 50 km/h (13.9 m/s)”, Please comment on the variations in the results as a result of changing the speed of the vehicle to some value other than 70 km/h.

15) In lines 149-150, “At least 7 complete passes of the sources were made for each experimental set-up.”, how does driving 7 times pass the source reflect back on the realistic conditions, at which there will be an orphan source? Please comment.

16) In lines 157-158, the ROI was selected with the prior knowledge of the source that you have used in your experimentation. Please comment how does a lost and unknown source would effect the results and the methodology that you have employed here.

17) In Eq. (3), please define $RD_i$.

18) It is understandable that you have considered the actual activity of the source to compute the degree of deviation in your results. Just to avoid confusion, please help add a statement that actual activity and position of the source does not need to be known when employing this method in realistic scenarios.

19) In lines 217-218, “the usual speed of the vehicle is about 50 km/h combined with acquisition intervals of 1 s”, please cite relevant papers to support this statement.

20) In line 234, the authors introduced “relative alignment”, I am confused with it, please explain clearly.

21) In lines 244-246, is there any physical reason behind this variation? Please explain briefly.

22) In line 260, “despite regardless”, remove “despite”, revise accordingly. Check the punctuation as well.

23) Regarding the computational resources and the programming language used, will it be possible to parallelize your method to enhance the computational speed? Please comment briefly.

24) In line 284, what do you mean by “adjacent measurement coordinates”?

25) In line 293-294, "…correct values of the recorded count rate would be assigned incorrect coordinates…", please re-write and rephrase.

26) Considering the results shown in Fig. 7, please explain why the SNR values for different detector and experiments is lower for Co-60 when compared to Cs-137?

27) I am confused about the trends of the lines shown for different quantile in Fig. 11. I am assuming parameters a and b are those used in Eq. (5), from the reported values shown in the figure, I guess there might be a mistake, unless I am misunderstanding something here.
Let us consider Fig. 11, SNR values keep increasing on the x-axis (i.e., only positive values), linear or log-scale. Using Eq. (5), and setting x from 1 to 150, for 50% curve you have reported a = -8.3 and b = 0.012. However, I guess the b value should also be negative for the curve to start increasing as shown in your figures. See Fig. 1 below.

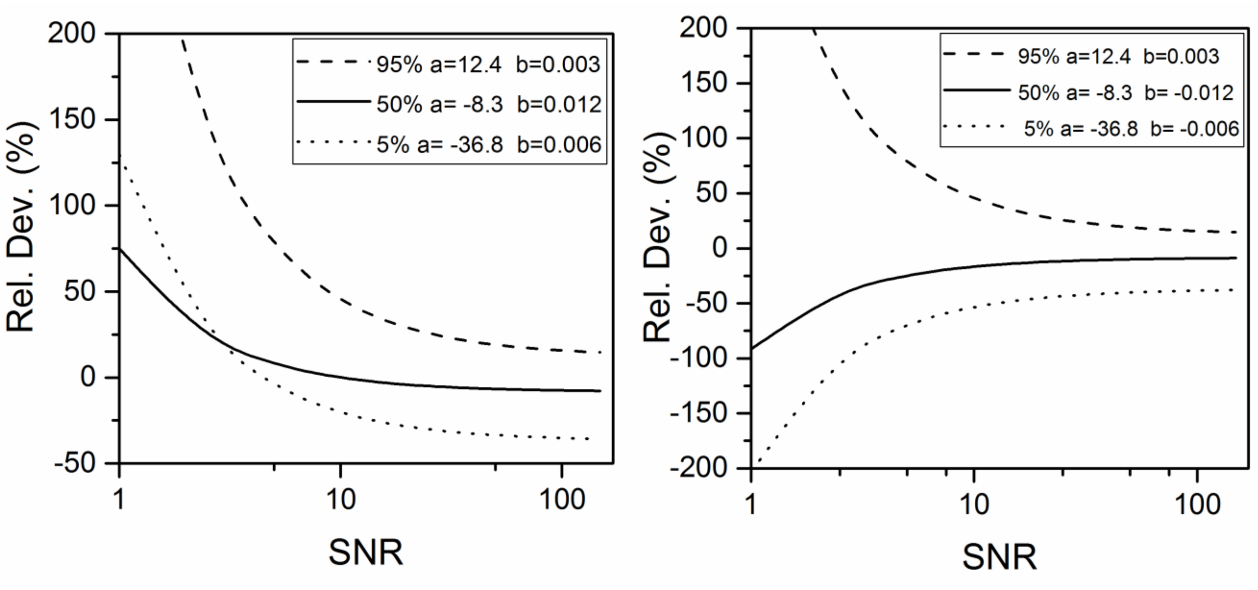


Fig. 1. Difference in the results based on changing b from positive to negative.

From your reported values, the plot should look as the left figure and it does not reproduce what you have shown in your paper. However, when considering b values negative for 50% and 5%, then we can reproduce your results. Please check your reported parameters carefully and revise accordingly. Same thing for another figure like Fig. 12, 13, 14 and 15, check all these and revise accordingly.

For your reference, here is the simple code for this test;

```
program main
implicit none
integer :: i,class
real*8 :: y,x,a,b
! compile with Gfortran
! command ->> "gfortran main.f90"
! "./a.out" to run the program
! choose the case 1, 2 or 3
! plot out.dat
open(100, file='out.dat')

print*, "enter case number (1), (2), (3): "
read*, class

select case (class)
case(1)
!this case if for 95% curve
a=12.4
b=0.003

case(2)
!this case if for 50% curve
a=-8.3
b=-0.012

case(3)
!this case if for 5% curve
a=-36.8
b=-0.006

end select

do i=1,150,2

x = real(i)

y= a+1/(b*x)

write(100,*) x, y
end do

end program main
```

28) The conclusion section is okay. However, please add some of the practical limitations that you have faced during your experimentation. It would be really useful for the readers to know all these.

# End of my comments
