# Supplementary material for: Accuracy of a Bayesian technique to estimate position and activity of orphan gamma-ray sources by mobile gamma spectrometry: Influence of imprecisions in positioning systems and computational approximations
Source: PLoS One. 2022 Jun 22;17(6):e0268556. doi: 10.1371/journal.pone.0268556 (PMC9216606; doi:10.1371/journal.pone.0268556)
Supplement: Attachment — Submitted filename: 2021 - PLOS ONE review - due July 4 2021.pdf [file pone.0268556.s002.pdf]

PLOS ONE review

Summary:

The authors present the results of a point source localization algorithm that uses Bayesian statistics to locate a source and estimate its activity. This paper is a follow-on paper to one from earlier this year (reference [13]), which gives more details of the Bayesian approach but only studied it with simulations; here they analyze the experimental results. The main conclusions they reach are that uncertainties in the detector positions strongly affect the source positions, and effects such as the finite sampling time and whether the point-source model is integrated over the sampling time also strongly influence the results for near-field sources.

The analysis of the data is thorough and fairly clear, especially given the complexity of the dataset being analyzed. The main weaknesses of this paper are twofold (and described in more detail below): (1) the analysis and conclusions are complicated by a unique data acquisition problem and (2) little to no context is provided for the results, even to the simulated results in [13], so it is difficult to evaluate the performance of the method.

Major comments:

(1) Handling of GNSS data issues

(1a) The authors find that their readout system exhibits what appears to be some kind of aliasing behavior in the GNSS spatial coordinates that leads to ~7-meter offsets along the direction of travel (line 282). These discrepancies are repeatedly referred to as “deviations” (lines 289-325), which is confusing with the usage immediately after of “relative deviation” as a key source localization metric. Please clarify the terminology.

(1b) They find a way to filter the data and locate the discrepancies, and then they notice that these discrepancies tend to repeat in groups of six. They create a model to generate positioning data with “synthetic deviations,” but the details are a bit murky (lines 301-307). Please clarify. (Also, in Figure 6 these are referred to as “simulated deviations,” which is confusing.)

(1c) The discrepancies in the bottom plot of Figure 6 look generally more sparse than those in the top figure. This figure is being used to justify the realism of the synthetic

deviation model, but it is not clear what quantitative comparison should be made by the reader.

(1d) The synthetic coordinates are used, along with synthetic gamma-ray data, to assess the impact of these discrepancies on the algorithm, and they are large (Figure 9). The section where the effect is assessed (lines 397-422) could possibly be moved here, otherwise it makes the rest of the results a bit disjointed.

(1e) The whole discussion of the coordinate discrepancies seems to beg the question of why the approach taken was not to filter out the bad points (as Fig 6 demonstrates) and then interpolate from the remaining points -- after all, the vehicle traveled at a remarkably constant speed. This conclusion would seem obvious from Fig 6, but it does not appear to be discussed, and would directly and perhaps significantly (for the better) affect the main results. Many lines are spent on what seems like a very complicated approach to a problem that seems like it could be solved with some straightforward data preprocessing.

(1f) My impression from reading this section is that the original, unfiltered coordinate data are used in the analysis, so the main results include the ~7-m offsets. Then it is not clear how any conclusions about the nearest sources can be made, such as the results in lines 373-396.

(2) Lack of context for the results

(2a) The results are lacking context to fully understand and evaluate the performance of the method. Since reference [13] performs simulations of what appears to be the same experimental setup with the same detectors, why are its results absent from this paper? It would be useful to see, e.g., that the experimental results are in line with the simulated expectation; or, if worse, what are the experimental factors contributing to the deficit?

(2b) In addition, no comparisons are made to other methods, such as maximum likelihood estimation or other Bayesian methods. There has been significant work in this field over the last decade and more, and it seems worthwhile to make some comparisons, both for context about how the source localization and activity quantification are performing, and for context about how this approach may or may not be less computationally intensive. At the very least, a maximum likelihood fit of equation (1) to time series count rate data, e.g., with r expanded to be sqrt((x-x0)^2 + y0^2), would be a useful comparison.

(2c) Along these lines, there are many other approaches one could discuss here in the introduction. A small subset of MLE and Bayesian approaches to this general problem includes:

- Rao, et al., “Identification of Low-Level Point Radiation Sources Using a Sensor Network,” 2008 International Conference on Information Processing in Sensor Networks.
- Deb, et al., “Radioactive Source Estimation Using a System of Directional and Non-Directional Detectors,” IEEE Transactions on Nuclear Science, 58 (2011).
- Jarman, et al., “Bayesian Radiation Source Localization,” Nuclear Technology, 175 (2011).
- Hite & Mattingly, “Bayesian Metropolis methods for source localization in an urban environment,” Radiation Physics and Chemistry, 155 (2019).
- Hellfeld, et al., “Gamma-Ray Point-Source Localization and Sparse Image Reconstruction Using Poisson Likelihood,” IEEE Transactions on Nuclear Science, 66 (2019).

Minor comments:

(3a) As noted two paragraphs after the equation, equation (1) is incomplete as-is. N-dot is not only a function of the scalar r, it is at least a function of the vector r since epsilon is not a constant but has a directional dependence. The background c is treated as a constant but that is also not necessarily true; it will have some time or spatial dependence completely unrelated to the source. It may be useful to clarify the equation.

(3b) Line 76: “After a detector has passed a radioactive source there will be a visible peak in the 76 measurement time series of the detector.” Wish that this were always the case, but at best this statement should say “may be visible,” due to the possibility that the source is not seen above counting statistics or that background variations mask the source.

(4) Lines 184-186: “the algorithm could not determine which side of the road the radiation originated from, thus resulting in probable positions of the source on both sides of the road, i.e., a bivariate posterior distribution of position of the source.” Should the term be “bimodal” and not “bivariate”?

(5a) The definition of SNR in (4) appears to be the same as is used in [13], but in [13] it is stated as clearly being calculated at the “maximum amplitude of signal”. Here it is

ambiguous whether it is calculated at the maximum amplitude of the signal or over a longer interval. Please clarify.

(5b) Small correction -- the symbol “I” in equation (4) is not the “total amplitude of the photopeak” but must be the sum of the background plus the photopeak, since B is subtracted from it.

(5c) Assuming that the SNR is calculated only at the maximum amplitude of the signal, since most (all?) source encounters span more than one single measurement, an alternate definition for SNR that might be worth using is the SNR integrated over an optimal time window -- e.g., finding N such that the N nearest spectra, denoting I_N as the counts I_N in the ROI of those spectra, that maximizes SNR(N) = (I_N - B*N) / sqrt(B*N). Since the algorithm is effectively able to integrate over time and not just use the single highest measurement, such a statistic should be more appropriate when analyzing its results.

(6) In the first Results section (lines 367-396), a comparison is made between a Cs-137 source at 10-m standoff and a Co-60 source at 20-m standoff. I assume this comparison was made because the two have similar SNRs, but those two isotopes will have different angular response functions, so it’s not exactly apples-to-apples. There are other Cs-137 measurements with somewhat similar SNRs (sites 5 and 6, at 20 and 40 meters). Those might make for a better comparison and stronger argument, by eliminating the difference in response function used.

(7) It seems like another effect not mentioned is the potential for parallax between the different detectors also causing difficulties localizing nearby sources. From [13] it appears that the two NaI detectors are offset from each other by at least ~1 meter, which will probably be a minor effect. Were the different detector positions considered when calculating the efficiencies used by the algorithm?

(8) It seems like some of the analysis difficulties (e.g., point versus integrated source count rates) could be solved just by having more frequent and accurate spatial coordinate and detector readouts. Can the authors comment on this point in the discussion? There are faster detector readouts available these days (even in list mode), and better positioning systems, like Inertial Navigation Systems (INS) or even Simultaneous Localization and Mapping (SLAM). What would they improve if they had the chance to retake this dataset and redo the analysis?

(9) What is the actual background rate B measured along the track, in counts per second? Does this rate vary over the measurement track, or is it consistent with a constant? Were measurements taken without sources to confirm the assumptions about background?

Copyedit comments:

- The first two sentences of the abstract are awkward and somewhat ungrammatical.
- There is frequent usage of “this” as a standalone noun. It is helpful to ask “this what?” to clarify these situations.
- Reference 15 should be “R Core Team,” not “Team RC”.
