# Supplementary material for: Accuracy of a Bayesian technique to estimate position and activity of orphan gamma-ray sources by mobile gamma spectrometry: Influence of imprecisions in positioning systems and computational approximations
Source: PLoS One. 2022 Jun 22;17(6):e0268556. doi: 10.1371/journal.pone.0268556 (PMC9216606; doi:10.1371/journal.pone.0268556)
Supplement: Attachment — Submitted filename: Answer_to_comments_2021-09-03.pdf [file pone.0268556.s003.pdf]

Reply to the comments:
The authors thank You for Your feedback and time. Hopefully, we have made all due corrections to the article to make it more understandable for the readers. Following are the answers to the questions.

1st reviewer:
Major comments:
(1) Handling of GNSS data issues
(1a) The authors find that their readout system exhibits what appears to be some kind of aliasing behavior in the GNSS spatial coordinates that leads to ~7-meter offsets along the direction of travel (line 282). These discrepancies are repeatedly referred to as "deviations" (lines 289-325), which is confusing with the usage immediately after of "relative deviation" as a key source localization metric. Please clarify the terminology.

Due to the importance of the "relative deviation" the term will be left as is. The positional "deviations" of the GNSS spatial coordinates will be referred to as "discrepancies" to avoid confusion as suggested. Manuscript altered accordingly.

(1b) They find a way to filter the data and locate the discrepancies, and then they notice that these discrepancies tend to repeat in groups of six. They create a model to generate positioning data with "synthetic deviations," but the details are a bit murky (lines 301-307). Please clarify. (Also, in Figure 6 these are referred to as "simulated deviations," which is confusing.)

The section has been rewritten. Hopefully, now it is more understandable for the reader.

(1c) The discrepancies in the bottom plot of Figure 6 look generally more sparse than those in the top figure. This figure is being used to justify the realism of the synthetic deviation model, but it is not clear what quantitative comparison should be made by the reader.

The purpose of the section was to display the potential effects of such discrepancies, possibly distorting the recorded data and subsequently reducing the quality of the data passed to the Bayesian algorithm. The aim was to raise awareness of those who might be looking forward to adopt the discussed methodology for orphan source search, without the intention of exactly replicating the mechanism of the discrepancies in this particular system with the utmost accuracy.

The section was amended accordingly to make this clear for the reader.

(1d) The synthetic coordinates are used, along with synthetic gamma-ray data, to assess the impact of these discrepancies on the algorithm, and they are large (Figure 9). The section where the effect is assessed (lines 397-422) could possibly be moved here, otherwise it makes the rest of the results a bit disjointed.

The suggestion was considered, but it was decided keep all of the results in the appropriate "Results" section.

(1e) The whole discussion of the coordinate discrepancies seems to beg the question of why the approach taken was not to filter out the bad points (as Fig 6 demonstrates) and then interpolate from the remaining points -- after all, the vehicle traveled at a remarkably constant speed. This conclusion would seem obvious from Fig 6, but it does not appear to be discussed, and would directly and perhaps significantly (for the better) affect the main results. Many lines are spent on

what seems like a very complicated approach to a problem that seems like it could be solved with some straightforward data preprocessing.

Indeed, it might be the case that using the post-processed spatial coordinates would provide a better result than using the raw experimental data in this exact case. But, because the aim of this Bayesian method is ultimately to be used in real-time for orphan source search, any additional post-processing of the data (or pre-processing before feeding the data to the algorithm) would impose another computational layer possibly reducing the real-time potential and increasing the complexity in applicability of the method to various mobile gamma spectrometry systems. Additionally, such post-processing algorithm would need to be very sophisticated to correctly deal with situations in real-time applications when the speed and direction of the vehicle is constantly changing. Furthermore, it might be that the discrepancies within the recorded coordinates could be minimized significantly without using any post-processing of the data, thus increasing the amount of the information passed to the Bayesian algorithm. But this is out of the scope of this paper.

The manuscript was edited accordingly to accentuate this information.

(1f) My impression from reading this section is that the original, unfiltered coordinate data are used in the analysis, so the main results include the ~7-m offsets. Then it is not clear how any conclusions about the nearest sources can be made, such as the results in lines 373-396.

To clear out any misconceptions – the filtering of the measurement coordinates done previously only filters out the portions of the data where the vehicle was turning around far away from the sources – i.e. certain measurement data and measurement coordinates are just being discarded. No other data manipulation was performed during filtering. Additionally, the filtering was only done while calculating the probabilities of the discrepancies discussed in section "Effects of the uncertainty in the coordinates of the measurement position on the Bayesian estimates".

Yes, it is correct that "raw", unaltered experimental data was used for the main results section. It is so, because the aim of this study was to investigate how well the developed Bayesian algorithm can predict the position and the activity of the source from the experimental data, which is usually affected by a variety of different factors introducing uncertainties in the recorded data. As mentioned previously, the main intention of this method is to hopefully use it in real-time, thus such discrepancies should either be minimized or just taken in to account.

(2) Lack of context for the results
(2a) The results are lacking context to fully understand and evaluate the performance of the method. Since reference [13] performs simulations of what appears to be the same experimental setup with the same detectors, why are its results absent from this paper? It would be useful to see, e.g., that the experimental results are in line with the simulated expectation; or, if worse, what are the experimental factors contributing to the deficit?

Comparison of relative deviations obtained using experimental with deviations obtained from theoretical data was added.

(2b) In addition, no comparisons are made to other methods, such as maximum likelihood estimation or other Bayesian methods. There has been significant work in this field over the last decade and more, and it seems worthwhile to make some comparisons, both for context about how the source localization and activity quantification are performing, and for context about how this approach may or may not be less computationally intensive. At the very least, a maximum likelihood fit of equation (1) to time series count rate data, e.g., with r expanded to be sqrt((x-x0)^2 + y0^2), would be a useful comparison.

While there are many ways to perform statistical inference, with the mentioned maximum likelihood estimation (MLE) being one of them, they might not be directly comparable. Although it is indeed possible to obtain the most probable position and activity of the source by performing MLE, the scope of the result will not be the same. For example – performing MLE by optimizing the likelihood function should yield the values of the parameters of interest with the highest probability. On the other hand, utilizing Markov chain Monte-Carlo (MCMC) like in this study, the result is the posterior distributions of the parameters of interest, which include the uncertainty of the parameters of interest. This, of course, comes at an additional computational cost.

Regardless of the additional computational cost, MCMC is readily utilized throughout many fields due to it's universality (references below). This flexibility of the MCMC has a penalty on the efficiency of sampling of the posterior distribution. And while it is indeed possible to increase the efficiency of the sampling by using something like Hamiltonian Monte-Carlo at the cost of universality and simplicity of the sampling algorithm, the comparison of well-established statistical inference tools within orphan source search is out of the scope of this current paper dealing with uncertainties pertaining experimental data.

Despite that, we thank you for pointing to a topic which can potentially result in an interesting study on it's own, which will be considered strongly for the future.

References:

Y. K. Ying, J. R. Maddison, and J. Vanneste, "Bayesian inference of ocean diffusivity from Lagrangian trajectory data," *Ocean Model.*, vol. 140, p. 101401, 2019.

R. Sachak-Patwa, H. M. Byrne, and R. N. Thompson, "Accounting for cross-immunity can improve forecast accuracy during influenza epidemics," *Epidemics*, vol. 34, no. July 2020, p. 100432, 2021.

H. Wu, Y. Chen, S. Li, and Z. Peng, "Acoustic impedance inversion using Gaussian metropolis–Hastings sampling with data driving," *Energies*, vol. 12, no. 14, pp. 1–15, 2019.

J. T. Fischer, A. Kofler, A. Huber, W. Fellin, M. Mergili, and M. Oberguggenberger, "Bayesian inference in snow avalanche simulation with r.Avaflow," *Geosci.*, vol. 10, no. 5, pp. 1–17, 2020.

H. Tran and K. Khoshelham, "Procedural reconstruction of 3D indoor models from lidar data using reversible jump Markov Chain Monte Carlo," *Remote Sens.*, vol. 12, no. 5, 2020.

Q. Yang, E. B. Pitman, M. Bursik, and S. F. Jenkins, "Tephra deposit inversion by coupling Tephra2 with the Metropolis-Hastings algorithm: algorithm introduction and demonstration with synthetic datasets," *J. Appl. Volcanol.*, vol. 10, no. 1, pp. 1–24, 2021.

L. Zhang *et al.*, "Efficient reliability assessment method for bridges based on Markov Chain Monte Carlo (MCMC) with Metropolis-Hasting Algorithm (MHA)," *IOP Conf. Ser. Earth Environ. Sci.*, vol. 580, no. 1, 2020.

M. Betancourt, "A Conceptual Introduction to Hamiltonian Monte Carlo," 2017.

(2c) Along these lines, there are many other approaches one could discuss here in the introduction. A small subset of MLE and Bayesian approaches to this general problem includes:

●●●●●*Rao, et al., "Identification of Low-Level Point Radiation Sources Using a Sensor Network," 2008 International Conference on Information Processing in Sensor Networks.*

Deb, et al., "Radioactive Source Estimation Using a System of Directional and Non-Directional Detectors," IEEE Transactions on Nuclear Science, 58 (2011).
Jarman, et al., "Bayesian Radiation Source Localization," Nuclear Technology, 175 (2011).
Hite & Mattingly, "Bayesian Metropolis methods for source localization in an urban environment," Radiation Physics and Chemistry, 155 (2019).
Hellfeld, et al., "Gamma-Ray Point-Source Localization and Sparse Image Reconstruction Using Poisson Likelihood," IEEE Transactions on Nuclear Science, 66 (2019).

Thank you for pointing to additional relevant background information. The introduction section has been altered to include the suggested references.

Minor comments:

(3a) As noted two paragraphs after the equation, equation (1) is incomplete as-is. N-dot is not only a function of the scalar r, it is at least a function of the vector r since epsilon is not a constant but has a directional dependence. The background c is treated as a constant but that is also not necessarily true; it will have some time or spatial dependence completely unrelated to the source. It may be useful to clarify the equation.

The theory section has been amended for clarification.

(3b) Line 76: "After a detector has passed a radioactive source there will be a visible peak in the 76 measurement time series of the detector." Wish that this were always the case, but at best this statement should say "may be visible," due to the possibility that the source is not seen above counting statistics or that background variations mask the source.

Indeed. Manuscript altered to reflect the information.

(4) Lines 184-186: "the algorithm could not determine which side of the road the radiation originated from, thus resulting in probable positions of the source on both sides of the road, i.e., a bivariate posterior distribution of position of the source." Should the term be "bimodal" and not "bivariate"?

Manuscript altered accordingly.

(5a) The definition of SNR in (4) appears to be the same as is used in [13], but in [13] it is stated as clearly being calculated at the "maximum amplitude of signal". Here it is ambiguous whether it is calculated at the maximum amplitude of the signal or over a longer interval. Please clarify.

Indeed, it is the same. The section discussing the SNR of the data was rewritten to avoid confusion.

(5b) Small correction -- the symbol "I" in equation (4) is not the "total amplitude of the photopeak" but must be the sum of the background plus the photopeak, since B is subtracted from it.

Indeed. As mentioned previously, the section has been rewritten to avoid confusion including this correction.

*(5c) Assuming that the SNR is calculated only at the maximum amplitude of the signal, since most (all?) source encounters span more than one single measurement, an alternate definition for SNR that might be worth using is the SNR integrated over an optimal time window -- e.g., finding N such that the N nearest spectra, denoting I_N as the counts I_N in the ROI of those spectra, that maximizes SNR(N) = (I_N - B*N) / sqrt(B*N). Since the algorithm is effectively able to integrate over time and not just use the single highest measurement, such a statistic should be more appropriate when analyzing its results.*

Thank you for suggesting an alternative and probably even better method for calculating the SNR values for this kind of data. But, to keep this study as directly comparable to the previous one as possible, the SNR definition probably should be kept as-is to reduce confusion of the reader.

Despite that, in future studies the suggested methodology for calculation of the SNR values will be strongly considered.

(6) In the first Results section (lines 367-396), a comparison is made between a Cs-137 source at 10-m standoff and a Co-60 source at 20-m standoff. I assume this comparison was made because the two have similar SNRs, but those two isotopes will have different angular response functions, so it's not exactly apples-to-apples. There are other Cs-137 measurements with somewhat similar SNRs (sites 5 and 6, at 20 and 40 meters). Those might make for a better comparison and stronger argument, by eliminating the difference in response function used.

As explained in the manuscript, the shorter the distance between the trajectory of the detector and the source for a given distance between the measurements, the greater the deviation of point estimates from the integrated estimates. Because the speed of the vehicle in the experiment was 50 km/h, the distance between the subsequent measurements was roughly 14 m. Although as you have mentioned, the response of the detector is somewhat different for different sources, it is of interest to compare the deviations in point estimates from the integrated estimates of the count-rate function in situations when the distance to the source is smaller (10 m) and larger (20 m) than the intercoordinate distance (14 m). For this purpose the set-up 1 suits the best.

Despite that, it was clarified in the manuscript, that the results might be affected due to somewhat different response of the detectors to the differences in the energies of the primary gamma photons.

(7) It seems like another effect not mentioned is the potential for parallax between the different detectors also causing difficulties localizing nearby sources. From [13] it appears that the two NaI detectors are offset from each other by at least ~1 meter, which will probably be a minor effect. Were the different detector positions considered when calculating the efficiencies used by the algorithm?

Yes. Individual positions of the detectors relative to the global navigation satellite system (GNSS) receiver placed on the roof of the cabin of the vehicle was taken in to account while both – evaluating the efficiency of the detectors and performing the Bayesian inference.

(8) It seems like some of the analysis difficulties (e.g., point versus integrated source count rates) could be solved just by having more frequent and accurate spatial coordinate and detector readouts. Can the authors comment on this point in the discussion? There are faster detector readouts available these days (even in list mode), and better positioning systems, like Inertial Navigation Systems (INS) or even Simultaneous Localization and Mapping (SLAM). What would they improve if they had the chance to retake this dataset and redo the analysis?

Naturally – the more and better information is presented to any statistical data analysis algorithm, the better the accuracy and precision of the predictions. Operating detectors in LIST mode would offer the most information about the radiation field at a cost computational resources due to individual registration of each photon. As suggested, something like INS could be a possible solution to the uncertainties in measurement coordinates pertaining the experimental data. But further investigations and experiments would be required to confirm the gains and practical aspects of using such system, as it can not be said for certain that better systems will have no flaws. Additionally, such systems would be significantly more complicated compared to a simple system using a single GNSS receiver, limiting the applicability of the method.

(9) What is the actual background rate B measured along the track, in counts per second? Does this rate vary over the measurement track, or is it consistent with a constant? Were measurements taken without sources to confirm the assumptions about background?

The background count rate in the area is relatively stable. Graphical illustration of one of the background surveys is illustrated in Fig. 1, where counts in a 4l NaI(Tl) detector is shown.

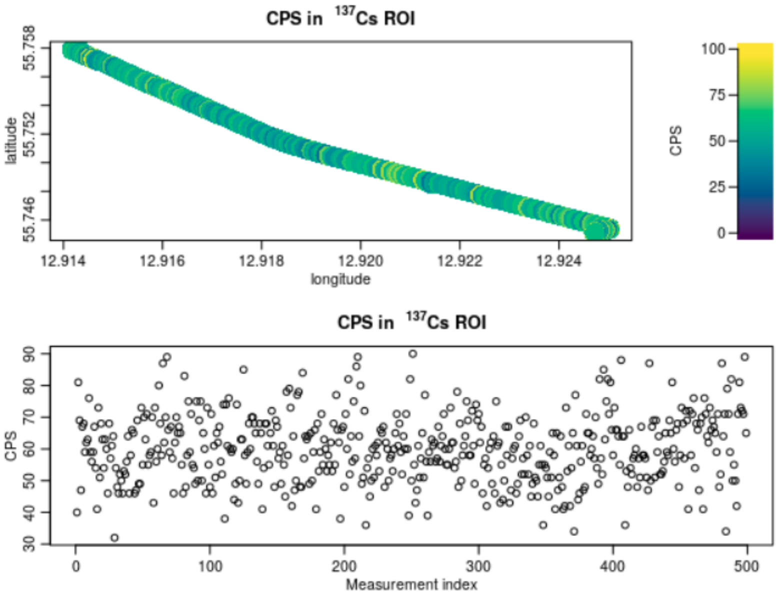

Fig. 1. Background count rate measurements in ^{137}Cs ROI along the whole trajectory.

Copyedit comments:
●●●The first two sentences of the abstract are awkward and somewhat ungrammatical.

Manuscript altered for clarification.

There is frequent usage of "this" as a standalone noun. It is helpful to ask "this what?" to clarify these situations.

Thank you for the advice!

Reference 15 should be "R Core Team," not "Team RC".

The mistake is now fixed.

2nd reviewer

General comment:
The present paper is the continuation of the author's previous work that was published in PLOS ONE. In this paper, authors investigated the accuracy of their proposed technique for estimating the activity and position of the source that has been lost. The present work is interesting and also important as it involves actual experiment to test the proposed theory. However, there are few issues that needs to be addressed. Please see my comments as attached and make changes to the current version of the manuscript. Considering my comments, I recommend minor revision of the manuscript before being considered for publication in PLOS ONE.
The comments:

1) In abstract, "This reduces the computational demands increasing the potential of real time applications of the method". Please re-write.

The sentence has been rewritten.

2) In line 9, "but gives no direct information on the distance to the source or its activity.", the detector is measuring some activity, it is better to refer to "actual activity" of the source, when addressing this.

Edited accordingly.

3) In line 15, "with which", consider changing to "at which".

Text was altered accordingly.

4) In line 47, "...orphan source the parameters of interest" add a comma after "source", please take care of the punctuation throughout the paper. There are many issues similar to what I pointed out here.

Text was altered accordingly.

5) In lines 58-59, "vague prior is usually used", do you mean something like an initial guess? Please revise accordingly.

Vague prior is a term used in Bayesian methods, assigning a probability distribution with a high variance to a parameter of interest, such that when multiplying the prior distribution with the likelihood to estimate the posterior distribution, the "broadness" of the uninformative or weakly-informative prior distribution allows the likelihood to be the determining factor in the calculations. This terminology is very well covered in Bayesian textbooks, for example Bayesian Data Analysis by Andrew Gellman et al.

Initial proposal of the MCMC chain is a totally different thing. It is the value(-s) of the parameter(-s) of interest, defining the starting location of the MCMC chain(-s). Then, during the MCMC iterations, the chain(-s) tend to converge to the locations of higher probability within the posterior distribution(-s). The starting location should have no effect on the posterior distribution, if the samples where the chain has been travelling to the higher probability region is discarded (this is regarded as using *burn-in* in Bayesian methods).

References:

Bayesian Data Analysis, Andrew Gelman, John B. Carlin, Hal S. Stern, David B. Dunson, Aki Vehtari, Donald B. Rubin, ISBN: 9781439840955, Chapman Hall/CRC, 2013

6) In line 59, "large variance", how do you defined this variance, statistically speaking "variance" would only be meaningful when we have a mean (refer to the formula). Please clarify briefly.

Indeed, it might be somewhat confusing to define a variance of a distribution. Despite that, if the distribution in question would be regarded as a collection of infinite number of random samples from that distribution, it would then be possible to estimate mean, median, variance or any other statistical measure of that distribution.

So, in essence, if a distribution with "large variance" would be randomly sampled, then the variance of these samples would also be large. There are analytical expression of variance of many distributions readily available. For example, the variance for a uniform distribution spanning from a to b can be calculated using $(b\text{-}a)^2/12$.

7) In lines 60-61, "However, if there is reason to believe that a radioactive source is in the area, it can be assumed that it could be anywhere in the area." Please re-phrase and re-write.

Text rewritten.

8) In line 64, "Similarly, a Gamma distribution with large shape and rate parameters...", this confusing to me, have you employed Gamma distribution in your model, or you just use this as an example since the distribution takes two parameters? Please clarify.

As explained in the theory section, Bayesian model is made up of two constituents – a prior and a likelihood. The prior is knowledge about the situation existing prior the measurements expressed in distributional form. So, Gamma distribution was used as a part of the prior for the activity of the sources in the Bayesian calculations.

9) In line 106, "a set of arbitrary initial values of", by "arbitrary" do you mean "guess values"? please revised accordingly.

Text revised accordingly.

10) In line 110, "uniform random number between 0 and 1", how did you generate these random numbers? Was it produced sequentially in your model or it's an array? How did the seed changes in each iteration? Please explain briefly.

The software package **R**, in which the Bayesian algorithm was written, has an array of default functions to evaluate distributions and draw random numbers in package "stats". For example, function *runif* was used to obtain continuously uniformly distributed pseudo-random numbers within a selected interval. System clock was used as the seed.

For details regarding the *runif* function please refer to Becker, R. A., Chambers, J. M. and Wilks, A. R. (1988) The New S Language. Wadsworth & Brooks/Cole.

11) In line 126, please define "GNSS".

It is now defined in line 46.

12) You have provided a schematic of the vehicle's path and source locations. It would be nice to

get some pictures of your experimental setup, so that the readers can understand how detectors were setup in the vehicle.

Picture added.

13) In Fig. 1, please consider changing the symbol for source location from "🎞" to something like a dot, as there will be lesser overlap between the symbols.

Although dots was considered at first, the crosses were chosen as a compromise. It is so because the dots have to be relatively large to ensure good visibility in variety of conditions and due to the sources being relatively close then some dots merge reducing the visibility of the data.

14) In line 149, "...at a steady speed of 50 km/h (13.9 m/s)", Please comment on the variations in the results as a result of changing the speed of the vehicle to some value other than 70 km/h.

Assuming that in the last part of the question "some value other than 50 km/h" was meant, the theoretical effect of the change of the speed is very simple – the higher the speed of the detector past the source, the less time spent in the vicinity of the source, the lower the quality and quantity of the information collected (lower number of counts) thus the more uncertain the estimates. Evaluating the actual effects of different survey speeds on the uncertainty of the Bayesian estimates is out of the scope of this particular paper as more investigations and experiments are needed, but will be considered for future publications.

15) In lines 149-150, "At least 7 complete passes of the sources were made for each experimental set-up.", how does driving 7 times pass the source reflect back on the realistic conditions, at which there will be an orphan source? Please comment.

Although there has been 7 or more passes of the sources was made in the experiment for every experimental set-up, Bayesian estimations were performed for each individual pass, in the text regarded as a segment. Text clarified to avoid confusion.

16) In lines 157-158, the ROI was selected with the prior knowledge of the source that you have used in your experimentation. Please comment how does a lost and unknown source would effect the results and the methodology that you have employed here.

In a case of unknown source, a list of ROIs for the expected orphan sources could be prepared beforehand. Then, leveraging the advancements in computational resources, a number of different MCMC chains could be launched for the whole list of ROIs using a strong multi-core processor to obtain estimations for all of the ROIs simultaneously.

17) In Eq. (3), please define Rdi.

Text ammended.

18) It is understandable that you have considered the actual activity of the source to compute the degree of deviation in your results. Just to avoid confusion, please help add a statement that actual activity and position of the source does not need to be known when employing this method in realistic scenarios.

Indeed. The manuscript has been altered accordingly to avoid confusion.

19) In lines 217-218, "the usual speed of the vehicle is about 50 km/h combined with acquisition

intervals of 1 s", please cite relevant papers to support this statement.

The section was ammended.

20) In line 234, the authors introduced "relative alignment", I am confused with it, please explain clearly.

If a gamma radiation detection system is configured to provide counts integrated over a certain acquisition time, it will output an integrated count-rate value after each successive acquisition time interval. If a detector is moving past a radioactive source at a set speed while the acquisition is being performed, the peak in the count-rate function (if present) can be portioned in to acquisition intervals of a set length in many ways, depending on the length of the acquisition interval and the relative alignment of the acquisition time intervals of a set length to the count-rate function, or more specifically – to the possibly present peak in the count-rate function after a passage of a radioactive source (as illustrated in Fig. 2).

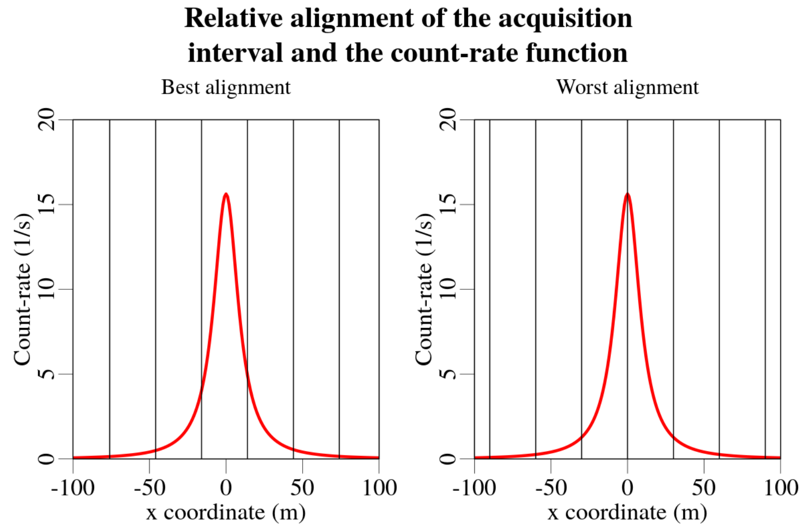

Fig. 2. Relative alignment of the acquisition time slots to the count-rate function. Graph on the left denotes the best alignment theoretically resulting in the biggest count-rate in a single acquisition time slot. Graph on the right illustrates the worst alignment, when the portion of the peak in the measurement time-series with the highest number of counts is divided between two adjacent measurement time-slots.

Different relative alignments will result in different maximum attainable counts during a passage of the radioactive source. In the best-alignment situation the maximum attainable counts in an acquisition time interval will be the highest, and in the worst alignment situation – the maximum attainable counts will be lowest. The difference between the maximum attainable counts in best and worst alignment cases depends on the relationship between distance to the source and the length of the acquisition time interval.

Text in the manuscript revised accordingly.

References:

Mobile search of material out of regulatory control (MORC) – Detection limits assessed by field experiments, NKS Technical Report NKS-421, 978-87-7893-510-6, R. Finck, T. Geber-Bergstrand, J. Jarneborn, G. Jónsson, M. Jönsson, S. Juul Krogh, S. Karlsson, J. Nilsson, M. Persson, P. Reppenhagen Grim, C.L. Rääf, M. Sickel, P. Smolander, R. Watson, K. Östlund

21) In lines 244-246, is there any physical reason behind this variation? Please explain briefly.

Clarified in the manuscript.

22) In line 260, "despite regardless", remove "despite", revise accordingly. Check the punctuation as well.

Manuscript altered accordingly.

23) Regarding the computational resources and the programming language used, will it be possible to parallelize your method to enhance the computational speed? Please comment briefly.

Due to the dependence of the new proposed sample on the previous sample it is hard to directly employ efficient parallelization within one MCMC chain. However, it is easily possible to run multiple MCMC chains at the same time with different initial starting parameters and seeds.

Manuscript altered accordingly

24) In line 284, what do you mean by "adjacent measurement coordinates"?

Considering recording the measurement coordinates for *i*-th measurement, "adjacent measurement coordinates" denotes the coordinates of the *i*-1 and *i*+1 measurements.

25) In line 293-294, "...correct values of the recorded count rate would be assigned incorrect coordinates...", please re-write and rephrase.

Manuscript altered accordingly.

26) Considering the results shown in Fig. 7, please explain why the SNR values for different detector and experiments is lower for Co-60 when compared to Cs-137?

Indeed, the SNR values for ^{60}Co are lower than ^{137}Cs. From Fig. 3. in the manuscript it is very easy to see that for the same experimental setup the distance to the ^{137}Cs source is from 10 to 30 metres closer for than ^{60}Co. This results in an effective difference in the distance for different sources for the same experimental setup in the interval of 20-100%, depending on the particular experimental set-up.

27) I am confused about the trends of the lines shown for different quantile in Fig. 11. I am assuming parameters a and b are those used in Eq. (5), from the reported values shown in the figure, I guess there might be a mistake, unless I am misunderstanding something here.
Let us consider Fig. 11, SNR values keep increasing on the x-axis (i.e., only positive values), linear or log-scale. Using Eq. (5), and setting x from 1 to 150, for 50% curve you have reported a = -8.3 and b = 0.012. However, I guess the b value should also be negative for the curve to start increasing
as shown in your figures. See Fig. 1 below.

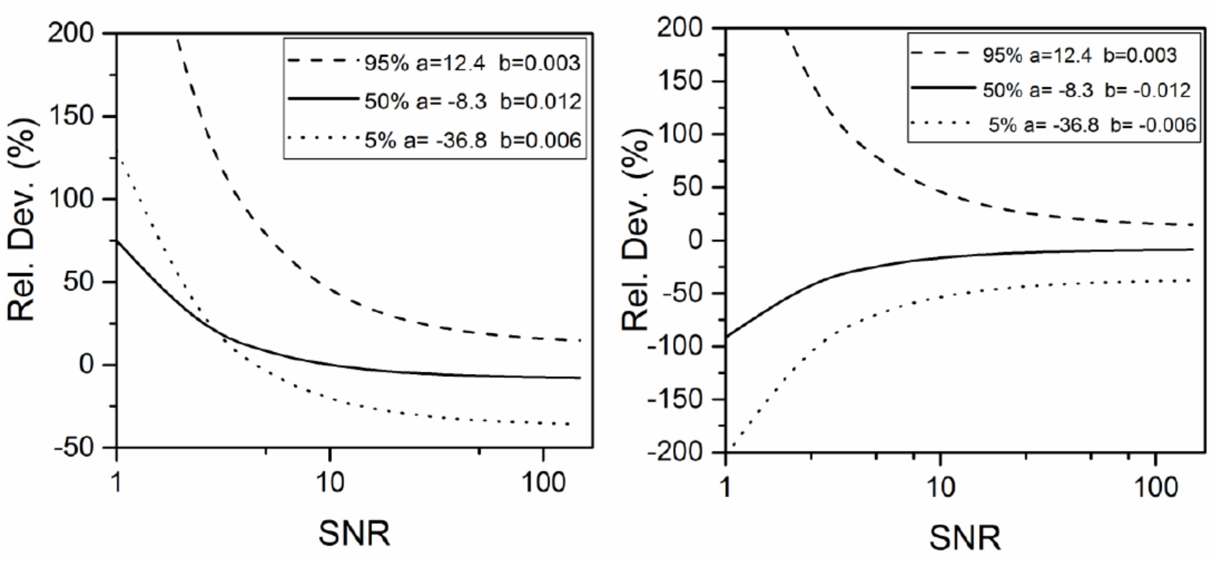

Fig. 1. Difference in the results based on changing b from positive to negative.
From your reported values, the plot should look as the left figure and it does not reproduce what you have shown in your paper. However, when considering b values negative for 50% and 5%, then we can reproduce your results. Please check your reported parameters carefully and revise accordingly. Same thing for another figure like Fig. 12, 13, 14 and 15, check all these and revise accordingly.

There was a mistake in the plotting procedures, which reported the incorrect values in the plots. As you have noticed, some of the *b* values had the opposite sign.

The procedures were fixed and the subsequent plots are now correct.

For your reference, here is the simple code for this test;

```
program main
implicit none
integer :: i,class
real*8 :: y,x,a,b
! compile with Gfortran
! command ->> "gfortran main.f90"
! "./a.out" to run the program
! choose the case 1, 2 or 3
! plot out.dat
open(100, file='out.dat')
print*, "enter case number (1), (2), (3): "
read*, class
select case (class)
case(1)
!this case if for 95% curve
a=12.4
b=0.003
case(2)
!this case if for 50% curve
a=-8.3
b=-0.012
case(3)
!this case if for 5% curve
a=-36.8
```

```
b=-0.006
end select
do i=1,150,2
x = real(i)
y= a+1/(b*x)
write(100,*) x, y
end do
end program main
```

28) The conclusion section is okay. However, please add some of the practical limitations that you have faced during your experimentation. It would be really useful for the readers to know all these.

A paragraph was added discussing limitations.
