# Supplementary material for: Accuracy of a Bayesian technique to estimate position and activity of orphan gamma-ray sources by mobile gamma spectrometry: Influence of imprecisions in positioning systems and computational approximations
Source: PLoS One. 2022 Jun 22;17(6):e0268556. doi: 10.1371/journal.pone.0268556 (PMC9216606; doi:10.1371/journal.pone.0268556)
Supplement: Attachment — Submitted filename: 2021-10-29 - PLOS ONE review (round 2).pdf [file pone.0268556.s004.pdf]

PLOS ONE review

To the authors:

These comments reference the numbering of my first-round comments, and in some cases extend the enumeration:

(1) GNSS data issues -- I appreciate the changes and explanations given. I have some more comments:

(1c) *"The purpose of the section was to display the potential effects of such discrepancies, possibly distorting the recorded data and subsequently reducing the quality of the data passed to the Bayesian algorithm. The aim was to raise awareness of those who might be looking forward to adopt the discussed methodology for orphan source search..."*

I appreciate not wanting to over-promise on the performance of the algorithm, but this raw data problem is particular to this system and others would very likely not experience it (see next point).

(1g) The main remaining weakness of the paper, as I see it, is as follows. The particular GNSS data problem being experienced here is both unique and surmountable -- the manuscript says they are "due to peculiarities in how Nugget is programmed" and shows how they can be removed. (Ideally this software issue would have been addressed before data collection.) A more universal type of position error, for example, would be Gaussian-like errors on the GNSS coordinates, and Figure 8 indicates an approximately 1-m error on the positions once the discrepancies are ignored. So it is not clear what a reader could conclude in general about the effect of position errors on this algorithm, since the particular manifestation of position errors is so unique and peculiar to this system. Given the clear ability to identify when this software issue occurs, it would be perfectly acceptable and justifiable to replace those points with interpolations and then analyze the cleaned data, and I suggest that be done. It would also streamline the paper quite a bit, but the main results would probably remain about the same.

(1h) This time I noticed a nomenclature issue, that the GNSS coordinate discrepancies are sometimes referred to as "uncertainties," which would imply the existence of error bars or some other kind of quantification of the expected difference in position from the true position, which does not seem to be the case. Please consider using a different term. "Inaccuracies" or "discrepancies" might be better.

(2a) Context for the measurements -- Thank you for adding the simulated results to Figure 18 for context, that adds a lot of information for the reader.

(2b) You missed my point (a suggestion to compare against other published orphan source localization methods), and I did not have to be convinced about MCMC as a method, but including the simulated results (point (2a)) provides sufficient context for this work.

(2c) I am not entirely satisfied with the use of the citations in the introduction. Part of the text that was added says “[the?] majority of the studies were considering either experimental data obtained at a very small scale (distances between the source and the detector in the order of metres using sources of low activity) [4–7],” but I think that is too narrow. There are other studies of localizing sources using vehicle-borne systems at >10-m distance scales, and also studies using airborne systems. Some other studies at a similar scale as this one that might be relevant citations are:

- Aage & Korsbech, “Search for lost or orphan radioactive sources based on NaI gamma spectrometry,” Applied Radiation and Isotopes, 58(1), 2003.
- Ziock et al., “Source-Search Sensitivity of a Large-Area, Coded-Aperture, Gamma-Ray Imager,” IEEE Transactions on Nuclear Science, 53(3), 2006.
- Miller, et al., “Adaptively Reevaluated Bayesian Localization (ARBL): A novel technique for radiological source localization,” NIM A, 784, 2015.
- Tandon, et al., “Detection of radioactive sources in urban scenes using Bayesian Aggregation of data from mobile spectrometers,” Information Systems, 57, 2016.

Please consider adding citations to other larger scale vehicle-borne studies like these, and rewording the discussion of these other studies and methods.

(3a) I am satisfied with how this point was addressed.

(3b) The phrase “there will be a visible peak in the measurement time series of the detector after the detector has passed a radioactive source” is still here despite mention of a revision.

(4) The term “bivariate” was still here (line 212) despite mention of its replacement with “bimodal.”

(5) I am satisfied with how this point was addressed.

(6) I am satisfied with how this point was addressed.

(7) I am satisfied with how this point was addressed.

(8) Thank you for the clarification of this point and the added discussion. I want to respond to one comment though:

“Despite the chosen methodology to reduce the uncertainties, such improved systems would be significantly more complicated compared to a simple system using a single GNSS receiver, limiting the real-time applicability of the method.”

These days smartphones can perform GPS localization stabilized by an IMU, which is essentially what an INS is doing, so it is not clear that this is correct.

(9) I am satisfied with the information about the background.

Other points I noticed on this re-read:

(10) Equation (2) -- Is the efficiency truly a function of (x_i, x_{i-1}, p)? It seems from [10] that it is a function of (x_i - x_{i-1}, x_i - p). Three arguments gives the impression that a 3-D response function is used, but it seems only 2-D, correct?

(11) Equation (2) -- The standard notation for the Euclidean or L2 norm automatically includes the square root in the definition of the ||x|| operation. Since the operation is not defined in the text it would be useful to use the standard notation, or give a quick explanation of what it means.

(12) Line 354 -- “Results of the comparison are displayed below.” -- it is not clear which figure is being referred to.

(13) Line 567 -- The algorithm is “quite slow.” How slow? The number of iterations is mentioned, but it would be good to mention the time per iteration, or just the overall time. How close is the algorithm to “real-time” operation?

(14) Data -- I saw the link to the data (https://github.com/SpontaneousFusion/AUTOMORC_Data), but what are NBL files and how can one open them? There does not seem to be any metadata or description;

please provide it. Ideally, data would be distributed in a universal file format such as CSV or HDF5.
