# Supplementary material for: Accuracy of a Bayesian technique to estimate position and activity of orphan gamma-ray sources by mobile gamma spectrometry: Influence of imprecisions in positioning systems and computational approximations
Source: PLoS One. 2022 Jun 22;17(6):e0268556. doi: 10.1371/journal.pone.0268556 (PMC9216606; doi:10.1371/journal.pone.0268556)
Supplement: Attachment — Submitted filename: Answer_to_comments_art3r2_2022-01-25.odt [file pone.0268556.s005.odt]

Dear Reviewer(-s),

Within this document you will find answers to your questions according to the order of the questions within the document. Comments expressing satisfaction to our previous answers will be marked by dark green text for ease of navigation within the document.

(1) GNSS data issues -- I appreciate the changes and explanations given. I have some more comments:

(1c) “The purpose of the section was to display the potential effects of such discrepancies, possibly distorting the recorded data and subsequently reducing the quality of the data passed to the Bayesian algorithm. The aim was to raise awareness of those who might be looking forward to adopt the discussed methodology for orphan source search…” I appreciate not wanting to over-promise on the performance of the algorithm, but this raw data problem is particular to this system for sparse image recand others would very likely not experience it (see next point).

It might be so, that this exact geographical data problem is unique for this system. Despite that, it is impossible to completely eliminate all of the uncertainties pertaining geographical coordinates of the experimental data. The type and severity of these uncertainties might indeed be very different for different systems. Despite that – it is still important for the potential users of such an algorithm to be aware of this.

(1g) The main remaining weakness of the paper, as I see it, is as follows. The particular GNSS data problem being experienced here is both unique and surmountable – the manuscript says they are “due to peculiarities in how Nugget is programmed” and shows how they can be removed. (Ideally this software issue would have been addressed before data collection.) A more universal type of position error, for example, would be Gaussian-like errors on the GNSS coordinates, and Figure 8 indicates an approximately 1-m error on the positions once the discrepancies are ignored. So it is not clear what a reader could conclude in general about the effect of position errors on this algorithm, since the particular manifestation of position errors is so unique and peculiar to this system. Given the clear ability to identify when this software issue occurs, it would be perfectly acceptable and justifiable to replace those points with interpolations and then analyze the cleaned data, and I suggest that be done. It would also streamline the paper quite a bit, but the main results would probably remain about the same.

Despite the attractiveness of the interpolation as a solution for the data problem, it is not entirely possible to use this method due to the fact that the deviations are occurring randomly. Upon closer inspection of the intercoordinate distance plot in Fig. 7, it is possible to see that the number of occurrences where the intercoordinate distance was increased and decreased is not the same, and one does not necessarily follow the other. Because of this, there might be an accumulated deviation in the reported coordinate from the actual position of the vehicle throughout the survey time. The longer the survey, the higher the probability for this accumulated deviation to occur. Thus, no two locations of the measurement points are certainly known, which is required if interpolation of the measurement data would be used.
Despite all that, interpolation was attempted to perform before the passing the data to the Bayesian algorithm. There were no visible improvements in the results. One example of posterior distributions for 137Cs source from experimental setup 5 is displayed in Fig. 1, below. The posterior distributions for source position look very similar for both – original and interpolated data. Using the original data, the algorithm found two possible locations for the source for both sides of the road, while using interpolated data the algorithm found only one possible location for the source (on one side of the road). In both cases, the location with the highest probability was on the opposite side of the road than the source was actually positioned in (actual position of the source is marked with dashed lines). The distance between the point of maximum probability within posterior distributions to the actual position of the source (in this instance mirrored to the other side of the road, marked by green crossed diamond) were 6.4 and 6.2 m for original and interpolated data respectively. The difference is negligible, in many ways.
The ratios of the activity value of the highest probability to the actual activity of the source were 1.58 and 1.41 for original and interpolated data respectively. Despite the ratio for the interpolated data being closer to 1, the width of the 90% of the posterior distribution for source activity is higher – 206 MBq compared to the width of the 90% of the posterior obtained using original data – 159 MBq.

Figure 1. Posterior distributions for position of the source (top) and activity of the source (bottom) for 137Cs source from experimental setup 5. Posterior distributions obtained using original, unaltered data is displayed on the left. Posterior distributions obtained using interpolated data displayed on the right. There is very little difference between the results for both data sets.

Despite the fact, that the data that we have is not perfect and that interpolating the coordinates does not yield a significant improvement, the algorithm functions as intended displaying robustness and usefulness even in situations when the data is not perfect.

(1h) This time I noticed a nomenclature issue, that the GNSS coordinate discrepancies are sometimes referred to as “uncertainties,” which would imply the existence of error bars or some other kind of quantification of the expected difference in position from the true position, which does not seem to be the case. Please consider using a different term. “Inaccuracies” or “discrepancies” might be better.
We agree with the reviewer that the term “uncertainty” can be misleading, especially if considering the output of a single constellation of spectrometer + GPS navigator. Our idea was to look at the potential discrepancies of a single system as “random sample” out of many flawed systems, and thus consider the GPS flaws as an innate attribute to combined sensor+GPS systems in general, generating, on average, a certain discrepancy. However, in order not to confuse the reader, we therefore use the suggested term “discrepancy” consistently throughout the manuscript.

(2a) Context for the measurements -- Thank you for adding the simulated results to Figure 18 for context, that adds a lot of information for the reader. (2b) You missed my point (a suggestion to compare against other published orphan source localization methods), and I did not have to be convinced about MCMC as a method, but including the simulated results (point (2a)) provides sufficient context for this work.


(2c) I am not entirely satisfied with the use of the citations in the introduction. Part of the text that was added says “[the?] majority of the studies were considering either experimental data obtained at a very small scale (distances between the source and the detector in the order of metres using sources of low activity) [4–7],” but I think that is too narrow. There are other studies of localizing sources using vehicle-borne systems at >10-m distance scales, and also studies using airborne systems. Some other studies at a similar scale as this one that might be relevant citations are:
Aage & Korsbech, “Search for lost or orphan radioactive sources based on NaI gamma spectrometry,” Applied Radiation and Isotopes, 58(1), 2003.
Ziock et al., “Source-Search Sensitivity of a Large-Area, Coded-Aperture, Gamma-Ray Imager,” IEEE Transactions on Nuclear Science, 53(3), 2006.
Miller, et al., “Adaptively Reevaluated Bayesian Localization (ARBL): A novel technique for radiological source localization,” NIM A, 784, 2015.
Tandon, et al., “Detection of radioactive sources in urban scenes using Bayesian Aggregation of data from mobile spectrometers,” Information Systems, 57, 2016.
Please consider adding citations to other larger scale vehicle-borne studies like these, and rewording the discussion of these other studies and methods.

The introduction has been amended according to the suggestions.  

(3a) I am satisfied with how this point was addressed.

(3b) The phrase “there will be a visible peak in the measurement time series of the detector after the detector has passed a radioactive source” is still here despite mention of a revision.

Indeed, but the sentence was rephrased. Within the original submission it was:
“After a detector has passed a radioactive source there will be a visible peak in the
measurement time series of the detector.”
which is not entirely correct, as was rightfully noticed. It was corrected to:
“Providing the radioactive source is of sufficient activity and close enough to the trajectory of the detector, there will be a characteristic “clock formed” shape of the  pulse rate in the measurement time series of the detector after it has passed a radioactive source (See fig. X).”

The sentence now is further amended to make it clearer for the reader.

(4) The term “bivariate” was still here (line 212) despite mention of its replacement with “bimodal.”

Thank you for noticing the error! It is now fixed.

(5) I am satisfied with how this point was addressed.

(6) I am satisfied with how this point was addressed.

(7) I am satisfied with how this point was addressed.

(8) Thank you for the clarification of this point and the added discussion. I want to respond to one comment though:
“Despite the chosen methodology to reduce the uncertainties, such improved systems would be significantly more complicated compared to a simple system using a single GNSS receiver, limiting the real-time applicability of the method.” These days smartphones can perform GPS localization stabilized by an IMU, which is essentially what an INS is doing, so it is not clear that this is correct.

Hopefully, the amended text will be clearer for the readers now.

Other points I noticed on this re-read:
(10) Equation (2) -- Is the efficiency truly a function of (x_i, x_{i-1}, p)? It seems from [10] that it is a function of (x_i - x_{i-1}, x_i - p). Three arguments gives the impression that a 3-D response function is used, but it seems only 2-D, correct?

Yes, the counting efficiency is two-dimensional. The argument xi denotes the i-th measurement location. By passing the i-th and  i-1-th measurement location it is possible to evaluate the direction of the vehicle. Then, by taking in to account the position of the source, which is the third argument, it is possible to evaluate the efficiency for that relative angle of incidence.
 

(11) Equation (2) -- The standard notation for the Euclidean or L2 norm automatically includes the square root in the definition of the ||x|| operation. Since the operation is not defined in the text it would be useful to use the standard notation, or give a quick explanation of what it means.

Notation within the Eq. 2 is now fixed.


(12) Line 354 -- “Results of the comparison are displayed below.” -- it is not clear which figure is being referred to.

Text has been clarified.


(13) Line 567 -- The algorithm is “quite slow.” How slow? The number of iterations is mentioned, but it would be good to mention the time per iteration, or just the overall time. How close is the algorithm to “real-time” operation?

Depending on the number of data points, it roughly takes 3-5 minutes (180-300 s) for the MCMC chain to complete 30000 iterations, which is usually enough to obtain good posterior distributions with 10000 of the first iterations discarded as burn-in. To be able to use the algorithm in real-time, the time of the Bayesian estimation should ideally have execution time of less than a second, a few orders of magnitude lower. This is now clarified in the text.

(14) Data -- I saw the link to the data (https://github.com/SpontaneousFusion/AUTOMORC_Data), but what are NBL files and how can one open them? There does not seem to be any metadata or description; please provide it. Ideally, data would be distributed in a universal file format such as CSV or HDF5.

An R package was developed for effortless reading of these files, called “IMP_NBL”, which is also available from the same repository. The repository will be soon updated with a newest version of the package.
