# Supplementary material for: Accuracy of a Bayesian technique to estimate position and activity of orphan gamma-ray sources by mobile gamma spectrometry: Influence of imprecisions in positioning systems and computational approximations
Source: PLoS One. 2022 Jun 22;17(6):e0268556. doi: 10.1371/journal.pone.0268556 (PMC9216606; doi:10.1371/journal.pone.0268556)
Supplement: Attachment — Submitted filename: Answer_to_comments_rev3.odt [file pone.0268556.s006.odt]

Dear reviewers,

Sincere thanks for such involvement and effort in further improving the article!

We hope that with this revision all of the remaining questions are answered, and that the article will soon be ready for publishing.

In the text below you will find copy-pasted questions for ease of reference and naviagion and our answers directly below the questions. The copy-pasted questions are displayed in italic.

(1g) Thank you for reanalyzing some of the data using my suggestion to interpolate the data to eliminate the discrepancies, and for commenting on this in the paper. However, seeing those 2D plots of the position posterior gives rise to some more questions. From the paper, specifically the references to a bimodal posterior, also Figure 11, and the discussion of the choice to “mirror” the source locations across the road, I was under the impression that the posterior was always fairly symmetric across the road. Figure 11 especially gives the impression that not only is the posterior symmetric, but deviations in the data do not strongly affect the symmetry of the posterior across the road. But both of these plots show posterior distributions that are strongly asymmetric, with peak probabilities in a 2-to-1 ratio (assuming a linear scale) for the original analysis, and peak ratios of greater than 9-to-1 for the interpolated track. Can you please (A) be clear what the spatial posterior distributions look like in practice, by perhaps (B) including a figure showing the 2D posterior distribution, and (C) explain in the paper why the posteriors can be so asymmetric, and (D) better justify the decision to mirror the results across the track, in light of this asymmetry.

	After the MCMC chain has started, if the initial samples were leading towards one side of the road, the chain might get stuck on that particular side of the road. This might happen irrespective of the particular data set or the chosen Bayesian model. To counter this possible “bias” of the algorithm, the discussed method of moving the MCMC proposal to the other side of the road each 1000 MCMC iterations was implemented. If the data and the model does not provide with enough information for the algorithm to choose a particular side of the road, then the posterior for the source position should have fairly equal amounts of relative probability on either side of the road.
	On the other hand, if the MCMC chain had equal possibilities to sample on either side of the road and there was additional information within the Bayesian model or the data itself that allows to deconstruct more information regarding the source position (for example the angular calibration of the counting efficiency of the detectors that was included within the Bayesian model), then depending on the amount of additional information the posterior distribution might have more or less relative probability for each side of the road, leading to asymmetric posterior distributions.
	Manuscript was altered accordingly to reflect this with an additional figure displaying four examples of posterior distributions for source position with varying distribution of probability between the two sides of the road within the posterior distributions for source position for the same source and experimental setup.

(14) Thank you for the clarification that the code to read the data is in the separate GitHub repository at https://github.com/. Please include a reference to this repository in the data repository (perhaps in the README.md file) since it is not easily located from the data repository, and the only metadata provided merely says “Within the archive you will find AUTOMORC experiment data.” You may also want to include a reference to your paper once it comes out.

	Thank you for the suggestion! The README.md file in the repository was altered accordingly.
